# Supplementary material for: The Systems Biology Research Tool: evolvable open-source software
Source: BMC Syst Biol. 2008 Jun 29;2:55. doi: 10.1186/1752-0509-2-55 (PMC2446383; doi:10.1186/1752-0509-2-55)
Supplement: Additional file 1 — SBRT Archive. An archive of the current version of the Systems Biology Research Tool. [file 1752-0509-2-55-S1.zip › sbrt-1.4.0/doc/developers_guide/api/sbrt/shell/io/fba/package-use.html]

Uses of Package sbrt.shell.io.fba


|  |  |  |
| --- | --- | --- |
| **Overview** \| **Package** \| Class \| **Use** \| **Tree** \| **Deprecated** \| **Index** \| **Help** | |  |
| PREV   NEXT | **FRAMES**    **NO FRAMES**     **All Classes** |  |


---


## **Uses of Package sbrt.shell.io.fba**

| Packages that use sbrt.shell.io.fba | |
| --- | --- |
| **sbrt.shell.io.fba** | Provides classes and interfaces for reading and writing files relevant to Flux Balance Analysis. |
| **sbrt.shell.mng.fba** | Provides classes and interfaces for creating Flux Balance Analysis process managers. |

Classes in sbrt.shell.io.fba used by sbrt.shell.io.fba

****ColumnOrFbaOptOutputFile****
  
          This class is used to write column-oriented output files
containing FBA optimization data.

****FbaOptOutputFile****
  
          This interface is used to represent FBA optimization
output files.

****RowOrFbaOptOutputFile****
  
          This class is used to write row-oriented output files
containing FBA optimization data.

****SerFbaOptOutputFile****
  
          This class is used to write output files
containing FBA optimization data as serialized maps.

Classes in sbrt.shell.io.fba used by sbrt.shell.mng.fba

****ColumnOrFbaOptOutputFile****
  
          This class is used to write column-oriented output files
containing FBA optimization data.

****FbaOptOutputFile****
  
          This interface is used to represent FBA optimization
output files.

****FluxIntervalInputFile****
  
          This class is used to read flux interval input files.

****RowOrFbaOptOutputFile****
  
          This class is used to write row-oriented output files
containing FBA optimization data.

****SerFbaOptOutputFile****
  
          This class is used to write output files
containing FBA optimization data as serialized maps.

---


|  |  |  |
| --- | --- | --- |
| **Overview** \| **Package** \| Class \| **Use** \| **Tree** \| **Deprecated** \| **Index** \| **Help** | |  |
| PREV   NEXT | **FRAMES**    **NO FRAMES**     **All Classes** |  |


---
